# Progress in immunoregulatory mechanisms during distraction osteogenesis

**DOI:** 10.3389/fbioe.2025.1665192

**Published:** 2025-08-25

**Authors:** Shiyu Huang, Aoran Zeng, Qing Yin, Qiming Yang, Bing Zou, Meiying He, Juehan Wang, Qi Pan

**Affiliations:** ^1^ Department of Orthopaedic and Reconstructive Surgery/Pediatric Orthopaedics, South China Hospital, Medical School, Shenzhen University, Shenzhen, China; ^2^ School of Basic Medical Sciences, Medical School, Shenzhen University, Shenzhen, China

**Keywords:** distraction osteogenesis, immunomodulation, bone regeneration, cytokines, biomaterials

## Abstract

Distraction osteogenesis (DO) is an endogenous bone tissue engineering technique that harnesses the regenerative potential of bone and has been widely applied in limb lengthening, bone defect repair, and craniofacial reconstruction. The DO procedure consists of three distinct phases: the latency phase, the distraction phase, and the consolidation phase, each characterized by unique biological processes. In recent years, increasing attention has been directed toward the role of the immune system during DO. Emerging evidence demonstrates that immune cells exhibit dynamic and temporally regulated changes throughout the different phases of DO. Moreover, immunomodulatory clinical interventions—such as the regulation of immune-related factors and the application of bioactive materials—are becoming promising strategies for optimizing DO outcomes. This review aims to summarize the temporal characteristics of immune regulation during DO, elucidate the functions and regulatory mechanisms of various immune cells involved, and explore the potential of immunomodulatory biomaterials, thereby providing novel insights for improving DO-based therapies.

## 1 Introduction

Distraction osteogenesis (DO) is an endogenous bone tissue engineering technique that leverages the bone’s intrinsic regenerative capacity. Its theoretical foundation is rooted in the “tension-stress” principle proposed by Dr. Ilizarov in the 20th century (GA, 1989 Feb; 1989 January). By applying a continuous, stable, and gradual mechanical traction force to osteotomized bone segments, DO activates regenerative signaling pathways, stimulates cellular proliferation and new bone formation, and simultaneously promotes the coordinated elongation of adjacent soft tissues such as muscles, fascia, blood vessels, and nerves (Sailhan, 2011; Dhaliwal et al., 2016; Liu Q. et al., 2022).

DO has been widely applied in clinical fields such as limb lengthening, bone defect repair, correction of skeletal deformities, and maxillofacial reconstruction (Winters R, 2014; Efunkoya et al., 2014; Sandhaus, 2021; Shakir, 2021). Compared with traditional surgical methods, DO offers several advantages, including minimal invasiveness, the elimination of the need for secondary bone grafting, and the simultaneous regeneration of bone and soft tissue. These features make DO a valuable strategy for addressing the limitations of conventional approaches in managing bone defects and deformities.

The DO procedure is typically divided into three sequential phases: the latency phase, the distraction phase, and the consolidation phase (Sailhan, 2011). The latency phase, which begins immediately after osteotomy and lasts for approximately one to 2 weeks, resembles the early stages of fracture healing. During this period, a local hematoma forms at the osteotomy site, initiating an acute inflammatory response that lays the foundation for subsequent bone regeneration (Kolar et al., 2010; Alman et al., 2011a; Marsell and Einhorn, 2011a; Loi et al., 2016b). The distraction phase is the core of the DO process, characterized by the gradual separation of bone segments—usually at a rate of 0.5–1 mm per day—to create a distraction gap. Under sustained mechanical traction, fibroblasts and osteoblasts within the gap are activated, leading to abundant extracellular matrix deposition and concurrent neovascularization (Choi et al., 2002). The consolidation phase, lasting several weeks to months, involves the progressive mineralization and maturation of the newly formed bone, culminating in the restoration of structurally and functionally competent bone tissue. During this phase, the local vascular network continues to develop, and the mechanical integrity of the bone is gradually reestablished.

Recent studies have highlighted extensive crosstalk between the immune and skeletal systems, giving rise to the emerging field of osteoimmunology. Following bone injury, the immune system mounts a rapid response, initiating inflammation to clear necrotic tissue and secreting various cytokines and growth factors that recruit and activate osteogenic cells. This pro-inflammatory phase is subsequently followed by an anti-inflammatory and tissue repair phase, ultimately enabling stable bone regeneration.

Given that DO relies heavily on the bone’s intrinsic regenerative mechanisms, its unique mechanical microenvironment profoundly influences immune cell dynamics and functional states. Studies have demonstrated that the immune response during DO exhibits tightly regulated temporal characteristics: in the latency phase, tissue injury activates the immune system, with pro-inflammatory M1 macrophages predominating; in the distraction phase, sustained mechanical stress modulates the immune milieu, promoting the transition from M1 to M2 macrophages and enhancing tissue repair; in the consolidation phase, inflammation markedly subsides, and M2 macrophages dominate, facilitating angiogenesis and bone remodeling. In recent years, the functional roles of immune cells—particularly macrophages—in DO have become a focus of intensive research. However, the contributions of other immune cell populations, such as T cells and neutrophils, remain incompletely understood. Building on these immunoregulatory insights, emerging therapeutic strategies have incorporated clinical interventions involving cytokines (e.g., IL-10 and TGF-β) and the development of immunomodulatory biomaterials for DO applications.

This review aims to systematically summarize the temporal characteristics of immune regulation during DO, elucidate the functions and mechanisms of various immune cells in bone regeneration, and explore immunomodulation-based clinical interventions and biomaterial strategies, thereby providing new perspectives and potential therapeutic targets to optimize DO outcomes.

## 2 Immunological phenomena in DO

### 2.1 Latency phase

Biologically, the latency phase of DO closely resembles the initial stage of fracture healing, both characterized by acute inflammatory responses. Following osteotomy, a hematoma rapidly forms at the local site, consisting of peripheral blood, intramedullary hematopoietic cells, bone marrow stromal cells, and various cytokines and growth factors. This environment provides essential biochemical and cellular substrates for immune cell recruitment and subsequent tissue regeneration (Marsell and Einhorn, 2011b).

Innate immune cells, such as neutrophils and macrophages, are rapidly recruited to the osteotomy site and exert dual regulatory roles. On the one hand, they phagocytose necrotic tissue and cellular debris, thereby maintaining local microenvironmental stability. On the other hand, they secrete a large number of pro-inflammatory cytokines, including IL-1, IL-6, and TNF-α, which enhance the inflammatory response to injury and prevent microbial invasion (Kon et al., 2001; Cho et al., 2007; Yu et al., 2024). Moreover, relevant studies have demonstrated that these pro-inflammatory cytokines not only promote the initial migration, proliferation, and differentiation of mesenchymal stem cells (MSCs) but also trigger the onset of neovascularization, thereby creating favorable conditions for the subsequent phases of distraction osteogenesis (Leibovich et al., 1987; Alman et al., 2011b; Kovtun et al., 2016; Graney et al., 2020; Mantsounga et al., 2022; Yang et al., 2022).

Simultaneously, the inflammatory microenvironment induces the release of various growth factors, such as TGF-β, BMPs, IGFs, and VEGF (Ai-Aql et al., 2008; Hvid et al., 2016). These factors are crucial in mediating the crosstalk between immune and osteogenic cells. Specifically, VEGF facilitates neovascularization, thereby improving local oxygen and nutrient supply essential for tissue repair (Pacicca et al., 2003). TGF-β, initially released by platelets, contributes to callus formation and regulates MSC proliferation, differentiation, and extracellular matrix synthesis. BMP-2 and BMP-4 levels increase during the early latency phase and are further enhanced by mechanical stimulation during the distraction phase, promoting chondrogenic and osteogenic differentiation (Ai-Aql et al., 2008; Vural et al., 2017; Sampath and Vukicevic, 2020). IGF enhances both cellular proliferation and osteogenic differentiation, playing a vital role in new bone formation.

Collectively, the latency phase of DO is a multi-layered and dynamic biological process involving hematoma formation, acute inflammation, immune cell recruitment and activation, and a coordinated release of cytokines and growth factors. Immune cells not only eliminate damaged tissue but also secrete signals that recruit MSCs and promote their osteogenic differentiation. This complex network of cellular and molecular events forms the biological foundation for successful bone regeneration during DO.

### 2.2 Distraction phase

The distraction phase, the core stage of DO, begins approximately 5–7 days after osteotomy and latency. During this period, an external or internal fixator is used to apply a controlled mechanical distraction force to the bone segments, inducing mechanical stress in the osteotomy gap and stimulating new bone formation. Biomechanical studies have demonstrated that this controlled traction enhances MSC differentiation into osteoblasts and promotes angiogenesis by activating relevant signaling pathways (Sharifpanah et al., 2016; Zhang et al., 2022).

It is noteworthy that the final healing outcome of DO is largely influenced by the distraction rate during this phase. Therefore, precise regulation of distraction parameters is of great clinical significance for optimizing the biomechanical properties of the newly formed bone tissue and preventing complications such as delayed union. Previous studies have demonstrated that a relatively high distraction rate (2.7 mm/day) can disrupt angiogenesis and inhibit osteogenic differentiation, whereas a low rate (0.3 mm/day) fails to sufficiently stimulate angiogenesis and may lead to premature mineralization of the callus (Li et al., 1997; 1999; Parihar et al., 2022). Currently, the standard clinical distraction rate is 1 mm/day, a protocol initially proposed by Ilizarov and subsequently supported by other animal studies (GA, 1989 Feb; 1989 January).

Furthermore, Fu et al. proposed a two-stage rate-varying distraction strategy based on computational modeling. This strategy involves applying a relatively low distraction rate (L: 1 mm/day) during the early phase, followed by a higher rate (H: 2 mm/day) during the later phase of distraction. Among the different protocols evaluated, the “low-to-high” combinations, such as L11H2 (11 days at 1 mm/day followed by 2 days at 2 mm/day) or L7H4 (7 days at 1 mm/day followed by 4 days at 2 mm/day), demonstrated faster bony bridging and greater bone formation compared with the conventional constant low-rate distraction (1 mm/day × 15 days). It should be emphasized that the conclusions of this strategy are primarily derived from computational model predictions and have not yet been validated in large-scale animal or clinical studies. Nevertheless, this dynamic adjustment protocol represents a promising alternative to the traditional constant-rate distraction approach, with the potential to shorten the overall treatment duration and reduce the risk of complications associated with prolonged external fixation (Fu et al., 2021).

A shift in the local immune microenvironment—from a pro-inflammatory to an anti-inflammatory profile—is essential for effective bone regeneration during the distraction phase (Tsukasaki and Takayanagi, 2019; Yang et al., 2022). Mechanical stress modulates the phenotype and function of various immune cells, including macrophages, T cells, and B cells. Notably, macrophages undergo polarization from the pro-inflammatory M1 to the anti-inflammatory M2 phenotype under mechanical stimuli. M2 macrophages secrete anti-inflammatory cytokines (e.g., IL-10, TGF-β) that promote angiogenesis and support MSC proliferation, migration, and osteogenic differentiation (Jetten et al., 2014; Zhang et al., 2020b; Liang et al., 2021; Cai et al., 2023; Kontogianni et al., 2025). T and B lymphocytes also contribute by producing regulatory cytokines and growth factors critical for bone remodeling (Miron et al., 2024). However, the precise roles of lymphocytes in the distraction phase of DO remain underexplored, warranting further investigation into their regulatory networks and temporal dynamics.

The mechanical stimuli also modulate cytokine expression. Pro-inflammatory cytokines such as IL-1 and TNF-α are downregulated during this phase, whereas IL-6 remains highly expressed, exhibiting mechanosensitive characteristics (Cillo et al., 2000; Wang et al., 2005; Cho et al., 2007; Chen et al., 2021). IL-6 serves dual functions: it enhances the recruitment of MSCs and endothelial progenitor cells (EPCs) via upregulation of chemokines such as MCP-1, and it directly promotes osteogenic gene expression in migrating cells, thereby accelerating bone matrix formation (Cho et al., 2007; Ando et al., 2014; Yang et al., 2022).

Mechanical stimulation further upregulates TGF-β expression, derived from osteoblasts, MSCs, and connective tissue surrounding the distraction gap. Recent findings suggest that M2 macrophage polarization may enhance TGF-β paracrine signaling (Mehrara et al., 1999; Yeung et al., 2002; Cai et al., 2023; Hu et al., 2024). TGF-β supports cell proliferation, ECM synthesis, and angiogenesis via Smad-dependent and non-Smad pathways (e.g., p38 MAPK, ERK) (Chen et al., 2012; Li et al., 2017).

In summary, during the distraction phase of DO, mechanical cues orchestrate the expression of pro- and anti-inflammatory cytokines and growth factors, modulating the immune milieu to favor bone repair. The transition of macrophages to the M2 phenotype, reduction of excessive inflammation, and activation of osteogenic and angiogenic pathways collectively create a favorable environment for bone regeneration.

### 2.3 Consolidation phase

Upon achieving the targeted bone length, distraction ceases, and the DO process enters the consolidation phase. At this stage, the regenerate bone exhibits a characteristic spatial gradient: a central unmineralized zone, an adjacent zone of initial mineralization, and a peripheral remodeling zone (Rachmiel et al., 2002; Percival and Richtsmeier, 2013; Yang et al., 2022). These regions collectively undergo progressive mineralization and structural remodeling, ultimately restoring biomechanical strength and biological stability.

Immunologically, this phase remains highly dynamic. M2 macrophages dominate and support mineralization by secreting anti-inflammatory cytokines and modulating osteoblast activity. Concurrently, regulatory T cells (Tregs) increase in number, suppressing Th1- and Th17-mediated inflammation and reducing pro-inflammatory cytokine levels to prevent excessive immune activation during tissue remodeling (Li J. et al., 2020; Liang et al., 2022; Yu et al., 2024).

The RANKL/OPG axis plays a central role in determining bone quality and remodeling outcomes. RANKL, secreted by osteoblasts and bone marrow stromal cells, promotes osteoclast differentiation and activity, whereas OPG acts as a decoy receptor that inhibits RANKL-RANK interactions, thus regulating bone resorption (Udagawa et al., 2021). A moderate level of osteoclastic activity is beneficial for removing low-quality woven bone and recruiting osteoblasts for lamellar bone formation. Studies show that the RANKL/OPG ratio peaks in the mid-consolidation phase and declines later due to increased OPG expression (Zhu et al., 2007). Early in consolidation, IL-1 and TNF-α are upregulated and synergize with RANKL to promote osteoclastogenesis. However, excessive RANKL expression may lead to pathological bone resorption. *In vitro* data demonstrate that M2 macrophages can inhibit RANKL and enhance OPG expression via IL-10 and TGF-β secretion, forming a negative feedback loop to restrain bone loss (Li L. et al., 2024).

This temporally regulated mechanism ensures a physiologic balance between bone resorption and formation. It facilitates the removal of immature bone and promotes the formation of mature lamellar bone, ultimately achieving functional bone regeneration.

**FIGURE 1:**
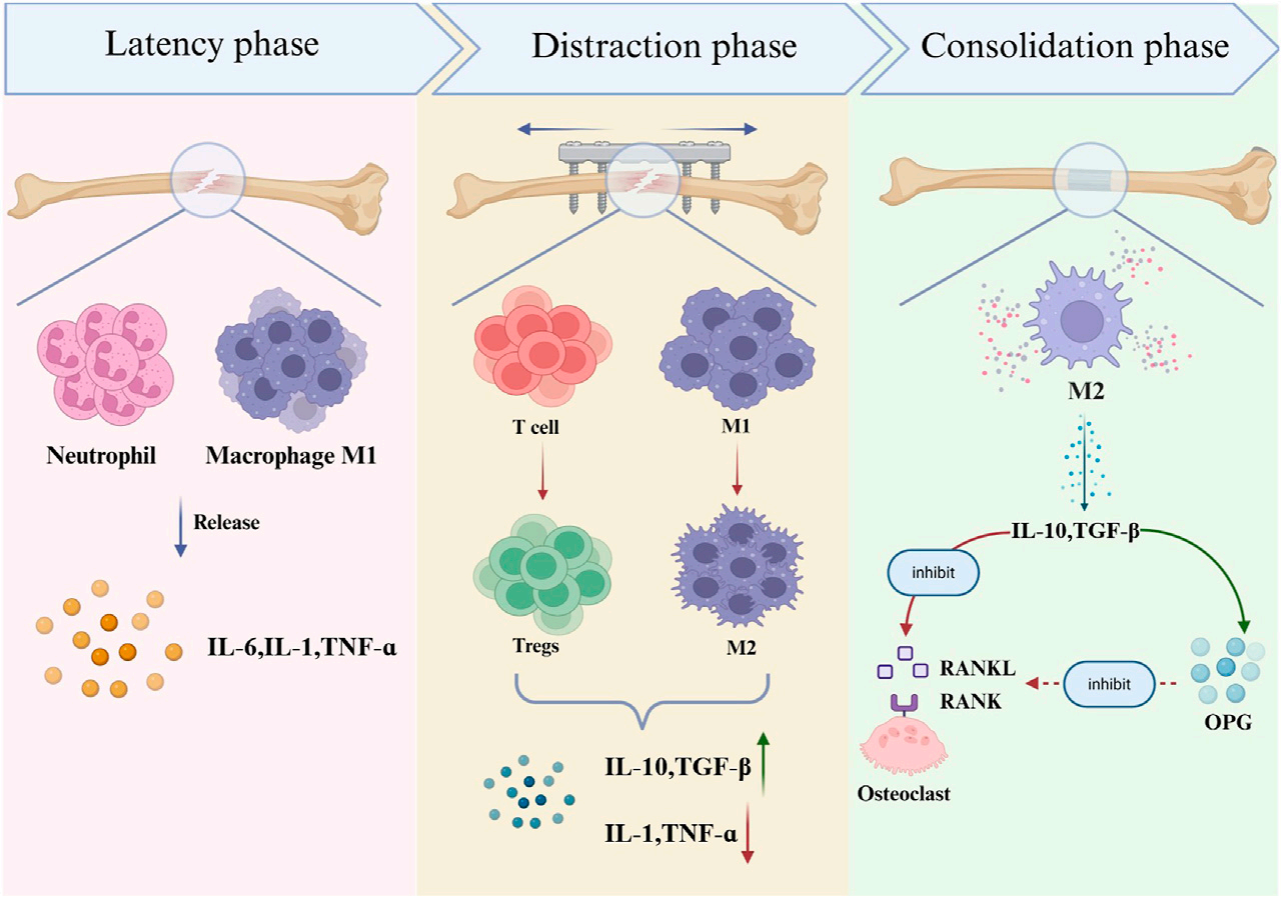
Immunological features during different phases of DO.

In the latency phase of DO, an acute inflammatory response occurs at the osteotomy site, with recruitment of innate immune cells such as neutrophils and macrophages. These cells secrete pro‐inflammatory cytokines including IL‐6, IL‐1, and TNF‐α. The distraction phase represents the critical period of DO, during which mechanical traction stimulates immune cells, including T cells and macrophages, to undergo phenotypic shifts from pro-inflammatory toward reparative states. Correspondingly, pro‐inflammatory cytokines decrease while anti‐inflammatory cytokines increase. During the consolidation phase, M2 macrophages dominate and secrete anti-inflammatory cytokines such as IL‐10 and TGF‐β, which inhibit the RANKL‐RANK interaction and promote OPG secretion. This regulation limits excessive bone resorption and ultimately facilitates functional restoration of bone architecture. The immunological events occurring during the different phases of distraction osteogenesis are illustrated in Figure 1.

## 3 Immune cells and bone regeneration during DO

### 3.1 Macrophages

As one of the most important innate immune cells, macrophages play multi-stage and multifunctional regulatory roles during DO (Chang et al., 2008; Pajarinen et al., 2019; Li L. et al., 2024). They exhibit remarkable heterogeneity and plasticity, capable of switching phenotypes and exerting distinct biological functions in response to changes in the local microenvironment. According to activation pathways, macrophages are generally classified into M1 and M2 phenotypes (Yunna et al., 2020; Miron et al., 2024). As previously described, M1 macrophages predominate in the early inflammatory phase (latency phase), secreting large amounts of pro-inflammatory cytokines such as TNF-α, IL-1, and IL-6, which mediate local inflammation, promote clearance of necrotic tissue, and initiate angiogenesis (Leibovich et al., 1987; Graney et al., 2020; Mantsounga et al., 2022). In contrast, M2 macrophages gradually become dominant during inflammation resolution and tissue repair phases (distraction to consolidation phases), secreting anti-inflammatory cytokines such as IL-10 and TGF-β, promoting recruitment, differentiation of osteoblasts, and matrix deposition, thereby facilitating new bone formation and remodeling.

#### 3.1.1 M2 macrophages promote bone regeneration

The early stage of bone regeneration is typically accompanied by pronounced inflammatory responses. While moderate inflammation is beneficial for initiating repair, persistent or excessive inflammation inhibits osteoblast activity and disrupts normal bone remodeling. Thus, the timely shift from pro-inflammatory to anti-inflammatory states locally is considered critical for successful bone regeneration. During this process, M2 macrophages secrete anti-inflammatory factors that regulate excessive inflammation, creating an immune microenvironment favorable for osteogenesis (Loi et al., 2016a). Notably, a recent study by Han et al. (Yao et al., 2025) revealed that exosomes derived from M2 macrophages can alter neutrophil maturation trajectories. By activating key reprogramming genes Acvrl1 and Fpr2, immature neutrophils are induced into an Anxa1-high (Anxa1^hi^) neutrophil subset with reparative phenotypes. These neutrophils communicate with MSCs and endothelial cells via the OSM signaling pathway, ultimately promoting bone formation and angiogenesis. This study unveiled a novel macrophage-mediated neutrophil phenotypic reprogramming mechanism, further elucidating the molecular pathways by which macrophages regulate bone regeneration.

Angiogenesis plays a pivotal role in bone regeneration. Newly formed blood vessels not only provide oxygen, nutrients, and a route for the clearance of metabolic waste in the bone repair region, but also serve as structural scaffolds and signaling cues for the recruitment and migration of various cells, including osteoblasts and BMSCs. This spatiotemporally coordinated process is referred to as the osteogenesis–angiogenesis coupling effect. It was recognized many years ago that, compared with M1 macrophages, M2 macrophages possess a stronger pro-angiogenic potential (Jetten et al., 2014; Hassanshahi et al., 2022). In recent years, increasing evidence has further elucidated the molecular mechanisms by which M2 macrophages participate in angiogenesis.

Wang et al. demonstrated that M2 macrophages strongly express SDF-1 (CXCL12) and VEGF, which recruit bone marrow–derived endothelial progenitor cells and promote their differentiation into CD31^+^ endothelial cells, thereby enhancing neovascularization and the formation of vascular structures (Wang et al., 2020). Isali et al. corroborated these findings and further clarified the mechanisms underlying the effects of M2 macrophages on endothelial cell proliferation, migration, and tube formation. Their results indicated that the M2^NECA^ subtype expresses higher levels of VEGF-A than other subtypes, and that VEGF-A can activate VEGF receptors on endothelial cells (e.g., VEGFR2), thereby triggering downstream PI3K/AKT and MAPK signaling pathways to promote endothelial cell proliferation and tubular network formation (Isali et al., 2025). Notably, multiple biological, physical, and chemical stimuli, such as BMP-2, trace Mg^2+^, and lactate, have been shown to promote M2 polarization by activating relevant signaling pathways, including pSmad1/5/8 and TLR-NF-κB, thereby enhancing VEGF secretion and forming a positive feedback loop that further stimulates angiogenesis (Wei et al., 2018; Zhang et al., 2019; Zhang et al., 2020a). In addition, growing attention has been paid to the role of exosomes secreted by M2 macrophages, which carry characteristic miRNA and regulate endothelial cell function via paracrine mechanisms (Yang et al., 2021; Lyu et al., 2022). For example, exosomal miR-21 derived from M2 macrophages can suppress PTEN expression in endothelial cells, thereby activating the PI3K/Akt–mTOR–HIF-1α–VEGF signaling axis and significantly enhancing endothelial cell proliferation, migration, and tube formation (Lyu et al., 2022). Similarly, Luo et al. reported that M2 macrophage–derived exosomes can promote angiogenesis by regulating the HIF1AN/HIF-1α/VEGFA pathway (Luo et al., 2024).

Bone marrow mesenchymal stem cells (BMSCs), possessing multipotent differentiation capacity, can differentiate into osteoblasts under a conducive microenvironment, playing a pivotal role in bone repair and reconstruction. Studies have shown that co-transfection of BMP-2 and TGF-β3 genes in rabbit BMSCs significantly enhances osteogenic differentiation through their synergistic effect (Wang et al., 2016). This suggests that M2 macrophage-secreted TGF-β and BMP-2 activate Smad and RUNX2 signaling pathways in BMSCs, promoting their osteoblastic differentiation and accelerating bone regeneration (Halloran et al., 2020; Sampath and Reddi, 2020; Mazziotta et al., 2021). Recent research further indicates that exosomes from M2 macrophages regulate BMSCs osteogenesis by delivering specific miRNAs. For instance, Zhang et al. found that M2 macrophage-derived exosomes enriched with miRNA-26a-5p significantly activate the osteogenic potential of BMSCs after uptake (Bin-Bin et al., 2022). Liu’s team also revealed that macrophage-derived exosomes promote BMSCs osteogenic differentiation via activating the BMP2/Smad5 signaling pathway (Liu et al., 2020). Wen et al. developed a biomimetic periosteum based on electrospun membranes and engineered exosomes, and demonstrated that this periosteum could promote the migration and osteogenic differentiation of BMSCs through the Rap1/PI3K/AKT signaling pathway, as well as enhance the secretion of VEGF by BMSCs to facilitate angiogenesis (Wen et al., 2025). Notably, ionic microenvironments also participate: Mg^2+^ modulates macrophage autophagy and polarization status, inducing secretion of exosomes enriched with miR-381, thereby establishing a new axis that promotes BMSC osteogenic differentiation (Zhu et al., 2022).

**FIGURE 2:**
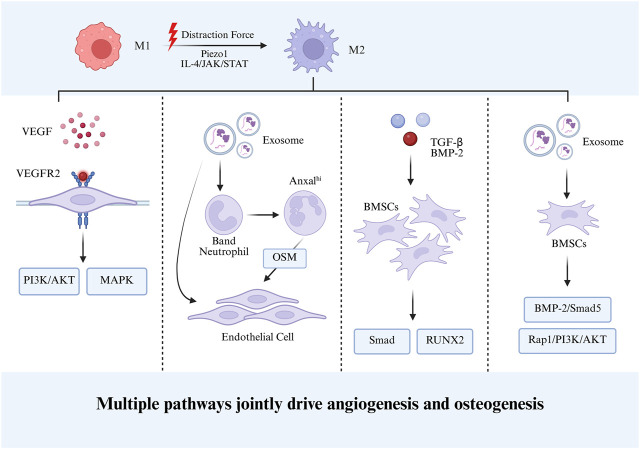
M2 macrophages promote bone regeneration during DO.

Collectively, by orchestrating immunoregulatory processes and tissue remodeling signals, M2 macrophages furnish indispensable cellular and molecular support for bone regeneration. This multifaceted activity underscores the pivotal role of immune–bone–vascular coupling in skeletal repair, thereby positioning M2 macrophages and their associated pathways as promising therapeutic targets for bone tissue engineering and regenerative medicine. Figure 2 depicts the mechanisms by which M2 macrophages promote bone regeneration during DO.

#### 3.1.2 Mechanical stimulation induces macrophage M2 polarization

Mechanical stress, as a key biophysical stimulus in bone tissue engineering, plays a regulatory role throughout the entire bone regeneration process. It has been demonstrated that mechanical stimuli not only directly promote osteogenic differentiation of MSCs but also indirectly enhance bone repair capacity by modulating the immune microenvironment. This provides a theoretical basis for regeneration strategies based on mechanical regulation such as DO.

At the cellular level, the regulatory effect of mechanical stress on osteogenic differentiation has been well established (Li Z. et al., 2020). For example, mechanical stress significantly upregulates osteogenesis-related gene expression in rat midpalatal suture chondrocytes (Kobayashi et al., 1999). More recent studies expand this understanding: Zhang et al. used 3D bioprinting to fabricate graphene composite scaffolds loaded with BMSCs, successfully inducing mature osteocyte-like 3D bone-like tissue under cyclic mechanical loading (Zhang et al., 2022). Another 3D-printed composite scaffold study confirmed that cyclic uniaxial compression stimulates BMSC osteogenesis while inhibiting peripheral blood monocyte-derived osteoclastogenesis, demonstrating bidirectional regulation (Kontogianni et al., 2025).

Recent research uncovers more complex mechanisms whereby mechanical stimulation promotes bone regeneration indirectly via inducing macrophage M2 polarization. Cai et al. demonstrated *in vitro* stretching and mouse treadmill running models that mechanical stress induces macrophage M2 polarization through the Piezo1 ion channel, promoting secretion of TGF-β1 and enhancing BMSC migration, proliferation, and osteogenic differentiation (Cai et al., 2023). Lu et al. found that mechanical stimulation activates the IL-4/JAK/STAT signaling pathway to induce M2 polarization, promoting tendon-bone healing (Liu Y. et al., 2022). Shao’s team innovatively showed that magnetic mechanical stimulation induces M2 polarization by activating integrin-associated cascades and suppressing MAPK/JNK pathways (Shao et al., 2023).

The hallmark of DO compared to conventional bone regeneration lies in its application of continuous, gradual mechanical traction. This dynamic mechanical environment not only directly activates osteogenic programs in BMSCs but, importantly, induces and sustains M2 macrophage polarization, forming a pro-regenerative immune microenvironment. As described, M2 macrophages secrete growth factors such as PDGF and VEGF to promote angiogenesis, while releasing BMP-2 and TGF-β to regulate osteoblast-osteoclast balance, serving as an “immune hub” in the DO process. This spatiotemporal coupling of mechanical stimulation and immune regulation positions M2 macrophages as key regulators linking mechanical signal transduction with tissue regeneration responses during DO.

During DO, persistent and progressive mechanical distraction stimulates macrophage polarization toward the M2 phenotype. On one hand, M2 macrophages secrete VEGF, which enhances endothelial cell proliferation and tube formation through the canonical PI3K/AKT and MAPK signaling pathways. On the other hand, M2 macrophage-derived BMP-2 and TGF-β promote the osteogenic differentiation of BMSCs via Smad and RUNX2 signaling pathways. In addition, exosomes secreted by M2 macrophages carry multiple miRNAs that facilitate neutrophil differentiation and activate multiple signaling cascades, thereby synergistically promoting angiogenesis and osteogenic differentiation.

### 3.2 Other immune cells

#### 3.2.1 Neutrophils

As a crucial component of the innate immune system, neutrophils have been a focal point in studies on bone regeneration. However, their precise role in bone repair remains controversial. Bastian et al. reported that a high-concentration neutrophil co-culture system inhibits mineralized matrix synthesis by BMSCs, evidenced by reduced cell numbers and significantly decreased ALP activity (Bastian et al., 2018). This finding was corroborated by Meesters et al., who demonstrated in a NOS knockout mouse model that excessive neutrophil infiltration in the callus tissue is significantly associated with nonunion phenotypes (Meesters et al., 2016). In contrast, recent studies have highlighted the positive roles of neutrophils in bone regeneration. Preclinical research using a rabbit cranial defect model showed enhanced bone regeneration in neutrophil-treated groups compared to controls (Herath et al., 2021). Mechanistically, IL-8 was found to promote neutrophil polarization toward the N2 phenotype, which via the SDF-1/CXCR4 axis activates PI3K/Akt and β-catenin signaling pathways, thereby facilitating BMSC homing and osteogenic differentiation (Cai et al., 2021). Notably, advances in biomaterials research have provided new evidence for modulating neutrophil function: Liu et al. developed a citrate-coordinated gelatin-chitosan hydrogel that enhances neutrophil-induced mesenchymal stem cell recruitment and osteogenic effects through histone acetylation modification (Liu et al., 2024). Moreover, neutrophil-derived exosomes, due to their unique bioactive components, have been shown to play critical roles in bone repair by promoting BMSCs osteogenic differentiation, angiogenesis, and cartilage regeneration through multiple pathways (Thomas et al., 2024; Wang L. et al., 2024; Wang et al., 2025).

It is worth noting that neutrophils respond to mechanical stimuli and function within the inflammatory milieu during the early stages of bone regeneration. Relevant studies have shown that mechanical stimulation activates the cation channel Piezo1, triggering calcium influx signals which, together with downstream Nox4 signaling, enhance neutrophil bactericidal capacity (Mukhopadhyay et al., 2024). However, research on how mechanical distraction influences neutrophil phenotype conversion and their regulatory mechanisms in bone regeneration remains limited, especially within the specific mechanical microenvironment of DO. Future studies should focus on key questions such as how mechanical signals regulate neutrophil polarization via mechanoreceptors like Piezo1; the impact of different mechanical loading patterns on neutrophil-mesenchymal stem cell interaction networks; and translational applications of neutrophil-mediated immunomodulation in bone tissue engineering. These research directions will provide a theoretical foundation for developing novel bone regeneration strategies based on immune-mechanical regulation.

#### 3.2.2 T Cells

Tregs, a functional subset of CD4^+^ T cells, possess potent immunosuppressive capabilities and are extensively involved in maintaining immune homeostasis, self-tolerance, and tissue stability. Recent studies have revealed that Tregs exert beneficial regulatory effects in tissue injury repair, particularly in bone regeneration.

Basic research indicates a protective role of Tregs in bone metabolism. Compared to wild-type mice, transgenic mice with elevated Treg levels exhibit increased bone mineral density and significantly reduced bone resorption (Zaiss et al., 2010). Schlundt et al. further demonstrated the pro-healing effects of Tregs in fracture repair. They confirmed that Tregs substantially improve bone healing quality and identified the ratio of effector T cells (TEFF) to Tregs as a key determinant of Treg therapeutic efficacy. Their results showed that exogenous Treg transplantation significantly promotes bone healing only when the preoperative TEFF/Treg ratio is below 0.07; otherwise, it is ineffective or may even delay the healing process (Schlundt et al., 2019).

Mechanistically, Tregs may protect bone tissue through multiple pathways. First, Tregs effectively suppress osteoclastogenesis, indirectly reducing bone resorption (Zaiss et al., 2007). Second, they modulate the local immune environment by downregulating pro-inflammatory cytokines such as IL-1β, IL-6, IL-17A, and RANKL, while upregulating anti-inflammatory factors like IL-10 and TGF-β, thereby mitigating inflammation-induced inhibition of bone formation (Han et al., 2018; Miron et al., 2024). Additionally, studies have shown that Tregs promote the assembly of the NFAT1-SMAD3 transcription complex in CD8^+^ T cells, driving Wnt10b expression, which acts on mesenchymal stem cells and osteoblasts to induce osteogenesis (Tyagi et al., 2018). Notably, a recent study reported that Treg induction is mechanosensitive and dependent on oxidative phosphorylation (OXPHOS), with higher matrix stiffness enhancing Treg induction and metabolic activity. This suggests that Tregs can sense mechanical changes in the tissue microenvironment and modulate immune responses to facilitate repair and regeneration (Shi et al., 2023).

In summary, Tregs serve as key immunoregulatory factors with significant pro-regenerative effects in bone healing. Although preliminary evidence supports their protective and osteogenic functions, the detailed mechanisms, especially in the context of DO, remain incompletely understood. Future research should further investigate the functional heterogeneity of Tregs in different bone disease models, their interactions with other immune cells, and optimize Treg-based therapeutic strategies to provide more effective immune modulation for clinical bone repair interventions.

## 4 Immuno-biological therapeutic strategies for DO

### 4.1 Immunomodulatory biomaterials and therapeutic approaches

Although DO s a clinically established technique in bone regeneration, its prolonged consolidation phase often entails pain, financial burden, and infection risks. Addressing these challenges, accelerating osteogenesis through adjuvant therapies has become a research focus. Based on the indispensable role of the immune system in regulating DO, novel therapies targeting the immune microenvironment to enhance osteogenic efficiency are emerging, offering innovative solutions beyond conventional DO.

IL-4, a classical M2 macrophage polarization factor, plays a key role in immune microenvironment modulation. Zheng et al. proposed an IL-4 delivery-based immunomodulatory strategy. In a rat cranial defect model, precise IL-4 delivery effectively orchestrated M1/M2 macrophage synergy, significantly promoting angiogenesis and bone regeneration (Zheng et al., 2018). Yang et al. developed a scaffold coated with mesenchymal stem cell membranes capable of inducing macrophage polarization toward the M2 phenotype, facilitating CD8^+^ T cell apoptosis and Treg differentiation, thereby accelerating bone healing (Su et al., 2023). Exosomes have demonstrated great potential in cell-free tissue regeneration therapies. Li et al. obtained exosomes from osteogenically pre-differentiated BMSCs and further encapsulated them in gelatin methacryloyl (GelMA), thereby achieving an “engineering-based” modification of the exosomes. These exosomes exhibited pronounced osteogenic and angiogenic properties and activated the p53 pathway via targeted miRNA delivery, reducing DNA oxidative damage and driving macrophage polarization from pro-inflammatory to anti-inflammatory phenotypes (Li X. et al., 2024). Similarly, Zhou et al. developed a biomimetic periosteum containing M2 macrophage-derived exosomes, which induced M2 polarization and promoted BMSC migration and osteogenesis through activation of the Rap1/PI3K/AKT pathway, markedly enhancing repair of large bone defects (Wen et al., 2025). Recent findings also indicate that intermittent parathyroid hormone injections increase macrophage numbers in DO new bone tissue and induce M1-to-M2 polarization by downregulating iNOS and upregulating Arg1, thus facilitating mandibular DO (Wang D. X. et al., 2024).

### 4.2 Other adjunctive therapeutic strategies

Beyond immunomodulation, recent studies have extensively explored molecular mechanisms and biomaterials applied in DO. These strategies enhance osteogenic differentiation and angiogenesis, providing novel avenues to optimize DO treatment. Table 1 summarizes representative recent studies and their mechanisms.

**TABLE 1:** Representative recent studies.

Interventions	Description	Mechanism
Bioactive Substances		
HOTAIR	long non-coding RNA	HOTAIR silencing enhances BMSCs osteogenesis via FTO degradation (Wu et al., 2025)
TP508	Thrombin peptide	Activates Wnt/β-catenin signaling to enhance BMSCs osteogenesis (Li et al., 2025)
TFRD	Total flavonoids of Rhizoma Drynariae	Activates PDGF-BB/VEGF/RUNX2/OSX axis to promote CD31hiEmcnhi vessel formation and angiogenesis–osteogenesis coupling (Zhao et al., 2023)
EGFL6	Epidermal Growth Factor-Like Domain-Containing Protein 6	Activates Wnt/β-catenin pathway to promote BMSC osteogenesis and angiogenesis (Shen et al., 2021)
ZN27	Small-molecule FAK activator (ZINC40099027)	Activates mechanosensitive FAK-ERK1/2 signaling, promoting endothelial migration, angiogenesis, and BMSC osteogenesis (Li et al., 2024b)
Biomaterials		
Co-MMSNs	Cobalt-doped mesoporous silica-coated magnetic nanoparticles	Enhance BMSC osteogenic differentiation and angiogenic gene expression (Zhao et al., 2023)
High-purity magnesium screws	-	Activate VHL/HIF-1α/VEGF signaling to enhance angiogenesis and osseointegration (Hamushan et al., 2020)
M-MSNs	Mesoporous silica-coated magnetic nanoparticles	Activate Wnt/β-catenin signaling to enhance MSC osteogenesis (Jia et al., 2019)
Therapeutic Strategies		
Hypoxic environment	-	Induces mesenchymal-to-epithelial transition (MET), activates Wnt/β-catenin signaling and autophagy, improving angiogenesis (He et al., 2020)
In vitro shock wave therapy	-	Mechanically stimulates osteoblast activity and angiogenesis (Ginini et al., 2018)
Percutaneous CO2 therapy	-	Improves local tissue oxygenation, promoting angiogenesis and osteogenesis (Kumabe et al., 2020)

## 5 Conclusion

In conclusion, this review systematically elucidates the critical regulatory roles of the immune system during distraction osteogenesis. Various immune cells, including macrophages, neutrophils, and T cells, are involved in a stage-specific and coordinated manner, influencing osteogenesis, angiogenesis, and tissue remodeling through the secretion of cytokines, exosomes, and modulation of intercellular interactions. Based on these immunoregulatory mechanisms, a range of novel biomaterials and adjunctive therapeutic strategies have been developed to modulate the local immune microenvironment and enhance the efficacy of distraction osteogenesis. Future studies should further investigate the immune–mechanical–skeletal coupling mechanisms and accelerate their translation into clinical applications.

## Abbreviations

DO, distraction osteogenesis; MSCs, mesenchymal stem cells; EPCs, endothelial progenitor cells; Tregs, regulatory T cells; BMSCs, bone marrow mesenchymal stem cells; ALP, alkaline phosphatase; NOS, nitric oxide synthase
